# Strength Development and Environmental Impact of Waste-Glass-Based Cements Activated with Portland Cement, NaOH, Na-Silicate or Na-Carbonates at Ambient Temperature

**DOI:** 10.3390/ma17205097

**Published:** 2024-10-18

**Authors:** Louise Lemesre, Rachida Idir, Martin Cyr

**Affiliations:** 1 Cerema, Univ Gustave Eiffel, UMR MCD, F-77171 Sourdun, France; louise.lemesre@univ-ubs.fr (L.L.); rachida.idir@cerema.fr (R.I.); 2 LMDC (Laboratoire Matériaux et Durabilité des Constructions), Université de Toulouse, UPS, INSA, 135, Avenue de Rangueil, F31077 Toulouse, France

**Keywords:** alkali-activated glass cullet powder, life cycle assessment, compressive strength, isothermal calorimetry

## Abstract

This paper presents an experimental approach to the study of the compressive strength, isothermal calorimetry and life cycle assessment (LCA) of alkali-activated pastes based on soda–lime–silica glass, established to investigate the effect of the nature and proportion of the activator. Four different activators are compared: Portland cement, sodium silicate, sodium carbonate (at four percentages by weight: 5, 10, 15 and 25 wt% relative to glass) and sodium hydroxide (3.5 wt%). Portland cement and sodium carbonate were added in dry form (powder), while sodium hydroxide (pellets) and silicate were used in solution. At room temperature, glass exhibited slow reaction kinetics, with mechanical performance increasing significantly beyond 28 days of curing. The nature of the activator had a direct impact on the mechanical performance of the activated glass. Cement-activated pastes and those containing 25 wt% of sodium carbonate developed strength at an early age (0–7 days). The other activators showed lower strength development before 28 days of reaction. While a higher activator content improved short-term performance, it also increased the environmental impact, primarily due to the activator. The LCA, conducted on 11 indicators, revealed that the environmental impact was largely driven by the type and amount of activator used. A performance impact indicator (PII) related to global warming was introduced to compare pastes with different performance values. At an early age (0–28 days), the PII was lower when the activator level was high but decreased over time as the strength improved. In terms of long-term performance (360 days), hydroxide and sodium carbonate (10 wt%) achieved compressive strengths of 91 and 74 MPa, respectively. These systems offered a balance between high performance and a reduced environmental impact, making them of interest for sustainable applications.

## 1. Introduction

A fraction of soda–lime–silica glass cannot be recycled to produce new glass products, which is usually only feasible when different colors of glass are recovered separately to produce manufactured glass products of the same color [1]. The collected glass is often mixed, making it unusable for the production of bottles of a specific color. Glass recycling, however, can reduce the volume of waste sent to landfills and lessen the dependence on natural resources of construction activities, particularly in civil engineering [2,3,4]. Several studies have explored the use of waste glass as aggregates or powdered glass as Supplementary Cementitious Materials (SCMs) in cement-based materials [5]. One advantage of using glass as an aggregate is that no additional grinding is required, though there is the potential risk of an alkali–silica reaction. This reaction, due to the high amorphous silica and alkali content in recycled glass, may cause expansion in concrete [1,6].

When glass cullet is ground into fine particles, it can be used as a partial substitute for cement in concrete [7,8,9,10,11]. According to the literature, incorporating glass powder into concrete can improve certain properties, such as durability [12,13,14]. The use of amorphous materials like granulated blast furnace slag (GGBS) in alkali-activated binders has been widely studied [15,16,17,18,19], and glass shares some properties with GGBS, such as its amorphous structure. Alkali-Activated Materials (AAMs) use aluminosilicate precursors (e.g., metakaolin or GGBS) activated with highly alkaline solutions. Some studies have used glass as a partial precursor mixed with more conventional precursor materials [20,21,22], or as the sole precursor in binders [23,24,25,26]. Pascual et al. [22] blended waste-glass powder (4400 cm^2^/g) and metakaolin as precursors for geopolymers and achieved a compressive strength of 30 MPa at 28 days (mortar with 8 wt% of metakaolin activated with NaOH, concentration at 5 mol/L). Puertas and Torres-Carrasco [27] studied a mixed activator solution of NaOH and Na_2_SiO_3_ to enhance the compressive strength of Glass Cullet Powder (GCP)-based mortars. Cyr et al. [23] demonstrated that soda–lime–silica glass could be effectively activated with NaOH and KOH solutions. These solutions, mixed with GCP at a fineness of 4170 cm^2^/g and cured at 40 °C, reached a compressive strength of 43 MPa at 28 days—comparable to that of common Portland cements. However, studies using glass as the sole precursor are rare due to its slow reaction kinetics and delayed strength development. Because glass is highly polymerized, it reacts more slowly than other commonly used precursors [28], which limits early-age strength in GCP-only cements. Zhang et al. [28] studied the heat release of different precursors: slag (GGBS), fly ash (PCFA Pet Coke Fly Ash) and glass powder (GP), all activated with NaOH solution (4 mol/L) with a liquid-to-solid ratio of 0.6. Their calorimetric study showed a significantly higher heat release from slag compared to the other precursors, particularly glass. The low intensity of glass’s heat flow curve reflects its lower reactivity. Liu et al. [29] also reported that the nature and alkali content of the activator are crucial factors for alkali-activated glass materials. Several studies have investigated the activation of glass powder with sodium hydroxide and have shown that a high concentration of activator can have a detrimental effect on the compressive strength. Cyr et al. [23] demonstrated that increasing the concentration of NaOH from 1 to 5 mol/L improved the mechanical performance of glass powder materials. However, a higher concentration (10 mol/L) led to a decrease in strength (Cyr et al. [23], Pascual et al. [22] and Rivera et al. [30]). In most studies where glass is used as the sole precursor, high temperatures (>40 °C) are systematically applied to achieve sufficient strength, particularly at early ages [20,29,30].

To summarize, the literature has shown that waste glass can be activated with several alkaline compounds or by applying heat. The advantages and disadvantages of each approach are outlined below.

- Strong bases (e.g., alkali hydroxides and alkali silicates) are effective at dissolving glass particles, but they are corrosive, which can make them difficult to use in certain practical applications. Sodium hydroxide, however, has the advantage of being less expensive and more widely available, making it one of the most commonly used alkali hydroxides in the synthesis of alkali-activated materials. The literature highlights the importance of controlling the amount of activator used to activate glass powder [22,23]. Studies show that excessive amounts of NaOH can be detrimental to the system; a study by Idir et al. [31] suggests that the optimal concentration is 3 mol/L.
- Liquid sodium silicate is known to be highly effective for activating blast furnace slags [32] and low calcium precursors such as fly ashes [25] and metakaolin [33]. Sodium silicates are generally defined by their SiO_2_/Na_2_O modulus, and the literature suggests that, for metakaolin and fly ash geopolymers, the preferred modulus range is 1.7–2.2 [34,35,36]. In the case of slags, lower moduli are sometimes used [37,38], referred to as metasilicates which, unlike higher modulus silicates, are in dry powder form.
- Alkali carbonates, which are less corrosive (though still potentially irritant), have not been as thoroughly studied. Due to their lower pH, strength development at early ages is slower [39,40]. However, several studies on slags [41,42] have shown that long-term performance with alkali carbonates is comparable to or even better than with alkali silicates. Furthermore, Duran et al. [43], demonstrated that using carbonate to activate slag results in shrinkage that is comparable to or less than other activators in cement-based mortars. As a result, the use of carbonates in alkali-activated materials has increased over the years. The reaction mechanism, particularly with blast furnace slag, has been explained by Bernal et al. [44]. In addition, the use of carbonate in powder form has enabled the development of a one-part, ready-to-use binder composed of dry material (precursor and activator), requiring only water to prepare the fresh mixture, similar to Portland cement-based materials.
- Thermal activation can improve the dissolution and reaction kinetics of glass [28,45], but its use is generally limited to prefabrication settings. It also increases both the environmental impact and the cost.

Although there is abundant literature on the activation of glassy compounds such as GGBS, selecting an appropriate activation system for soda–lime glass precursors remains challenging. To address this, an evaluation of four different systems, used at various proportions, is proposed in this paper: Portland cement, high modulus sodium silicate, sodium hydroxide and sodium carbonate. The selection is based on both engineering performance (compressive strength) and environmental impact. The environmental assessment of these systems, often overlooked in similar studies, is also considered here, as it is a key factor in ensuring the sustainable use of alkali-activated materials. No thermal activation was used for the systems in this study, and they were maintained at a constant temperature of 20 °C.

Portland cement is generally not used as an activator in alkali-activated binders. However, in this study, it serves as a reference for comparison with other activators. The reaction of Portland cement produces C-S-H gel, which provides initial cohesion while releasing alkali and calcium into the solution. This helps maintain the pH needed to attack the silica network of the precursor. In addition, using cement allows for the production of a binder composed solely of dry materials, offering the advantage of being a “classic” system—i.e., a combination of powders (cement and glass powder) and water. It must be used in moderate proportions to stay within the domain of alkali-activated materials and to avoid increasing the environmental impact. The aim of this study is to evaluate the efficiency of different types of activators for glass cullet powder, specifically Portland cement, sodium silicate, sodium carbonate and sodium hydroxide, and to compare their effects on both mechanical performance and the environmental impact of the resulting alkali-activated materials. This research seeks to provide insight into how the nature and proportion of activators influence the compressive strength development over time, as well as their sustainability in terms of life cycle assessment, particularly through the lens of global warming potential.

## 2. Materials and Methods

### 2.1. Materials

For this study, mixed-color bottle glass from municipal waste collection was first cleaned of residual organic matter, and then crushed and ground in a ball mill to a fineness of 3000 (±50) cm^2^/g (Blaine, according to NF EN 196-6 [46]). The particle size of the glass powder, obtained using laser granulometry, is presented in Table 1. The density was 2.5 g/cm^3^. Table 2 shows that the glass was mainly composed of silica (71.5 wt%), with 12.6 wt% of alkalis (Na_2_O_eq_ = Na_2_O + 0.658K_2_O), calcium (10.4 wt%) and a smaller quantity of alumina (2.3 wt%).

The CEM I 52.5N-type (according to EN 197-1 [47]) Portland cement was supplied by Calcia Ciments. The specific surface area, determined using the Blaine method, was 3850 cm^2^/g, and its density was 3.11 g/cm^3^. The chemical composition is given in Table 2 and the mineralogical composition was: 66% C_3_S, 10% C_2_S, 11% C_3_A and 8% C_4_AF.

The sodium hydroxide (NaOH) was supplied by Honeywell in pellet form at 98% purity. The alkaline solution of 3 mol/L was prepared by dissolving pellets in distilled water at room temperature. Previous studies [31] have shown this concentration to be the optimum.

The sodium silicate came from Woellner, under the name of Betol 39 T. It was composed (weight %) of 27.5% SiO_2_, 8.3% Na_2_O and 64.2% water. The molar modulus of this solution (SiO_2_/Na_2_O) was thus 3.42 and allowed a pH of about 11 to be obtained. This high modulus was chosen in order to reduce the corrosive impact of the activator, since the pH was around 11 rather than around 14 for the 1.7–2.2 modulus silicates. This had an impact on the quality of the activation, since lower pH values are known to greatly reduce the attack on the silica lattice of the precursors.

The sodium carbonate (Na_2_CO_3_) that was used (31432 Fluka from Honeywell) was packaged in powder form (99% purity). The sodium carbonate powder was added directly to the glass powder.

### 2.2. Methods

#### 2.2.1. Preparation of Activated Glass Pastes

The pastes were prepared using a water-to-solid ratio (w/s) of 0.30. The term “solid” refers to the glass powder and the activator (and the fraction of the sodium silicate that was not water). The glass powder was added to the activator solution (sodium hydroxide or sodium silicate) in the mixing bowl. In the case of Portland cement or sodium carbonate activation, the dry activators were mixed with the glass powder and then the water was added into the bowl. The pastes were mixed for 2.5 min before being poured into a set of cubic molds of 20 mm per side and tapped on a flat surface manually to release any residual air bubbles. The molds were covered to minimize moisture loss and stored at 20 °C. After 24 h, the samples were demolded, sealed in plastic bags and then cured at 20 °C until tested.

#### 2.2.2. Compositions of Activated Glass Pastes

Table 3 shows the proportions of the various constituents of the systems and the nomenclature used. For Portland cement with sodium silicate and sodium carbonate activation, four contents were tested: 5, 10, 15 and 25 wt% relative to glass, in order to study the effect of activation concentration on glass reactivity. As an example, the pc_5_ system corresponds to the system containing 5% by mass of CEM I 52.5N in relation to the glass powder. For sodium silicate activation, the total amount of water included the mixing water and the water contained in the sodium silicate used (64.2 wt%). Thus, the term “solid” referred to the glass powder and the dry matter in the sodium silicate solution (35.8 wt%).

For sodium hydroxide activation, the concentration of 3 mol/L NaOH was chosen to activate the glass powder, corresponding to a quantity of 3.5% of the total mass of the glass powder (sh3.5).

A paste was composed of only Portland cement (100 wt%) and referred to as pc_100_. This system was used as reference for the calorimetric measurement for systems activated with Portland cement.

#### 2.2.3. Testing Methods

The compressive strength was measured at curing ages from 1 to 360 days on a compressive testing machine working at a rate of 2.4 kN/s. Each compressive strength result was the mean value of 5 or 6 individual tests. Due to their small size, the samples measuring 20 × 20 × 20 mm may exhibit higher errors compared to larger mortar samples measuring 40 × 40 × 160 mm. Isothermal calorimetry (TAM Air 8 channel) was used to study the kinetics of the reaction of the fresh pastes at 20 ± 1 °C. Fresh pastes were first mixed externally and then poured into ampoules and weighed. The ampoules were then placed in the calorimeter after a stabilization time of 30 min and the heat flow was recorded for the first 7 days of the reaction. The results were normalized using the total weight of the paste.

**Figure 1:**
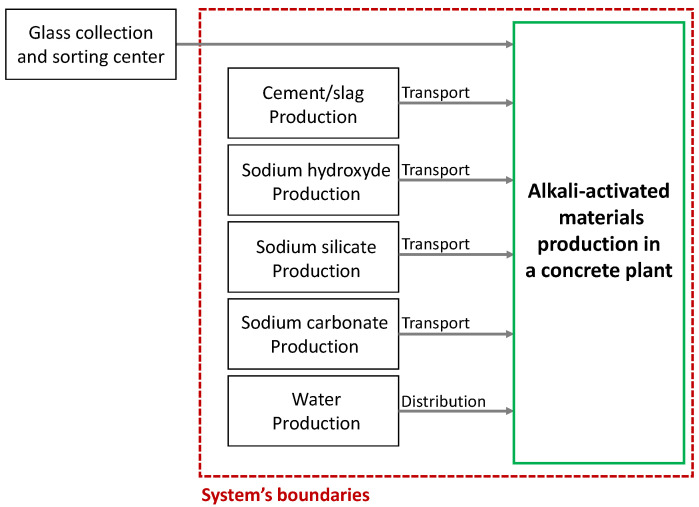
System boundaries for manufacture of alkali-activated materials.

The life cycle assessment method was used to analyze the environmental impact of the mixtures. The scenarios were modeled using the SimaPro 9.3.0.2 program, associated with the environmental database, Ecoinvent version 3.8 (2021). The CML method [48] was used to evaluate eleven different indicators of impact: abiotic depletion (kg Sbeq), energy consumption (MJ), global warming (kg CO_2_eq), ozone layer depletion (kg CFC-11eq), toxicity to humans (kg 1,4-DBeq), fresh water aquatic and marine aquatic ecotoxicity (kg 1,4-DBeq), terrestrial ecotoxicity (kg 1,4-DBeq), photochemical oxidation (kg C_2_H_4_eq), acidification (kgSO_2_eq) and eutrophication (PO_4_^3−^eq). The functional unit studied was “manufacturing 1 m^3^ of alkali-activated material”. Figure 1 shows the boundaries of the system concerning the production of constituents (cement, sodium hydroxide, sodium silicate, sodium carbonate, glass and water) and their transportation.

Table 4 summarizes information about the data used in this study: process, modulus used and data source assumptions. When the module does not exist in the Ecoinvent database, the process is modeled taking some assumptions into account. The glass cullet powder was considered a by-product, so only grinding and transport were taken into account. The glass fines were produced by grinding, lasting 8 h (for approximately 13 kg) in a ball mill. The power of the crushers considered in this study was 0.0262 kw/kg, which is an average of industrial ball mill data (min = 0.0177 kW/kg and max 0.0346 kW/kg). The sodium hydroxide used in this study was an undiluted product. The data (inputs–outputs) of the 50% diluted product from the Ecoinvent database were used, multiplied by two (×2). The French (75–80% nuclear) energy mix was taken into account when electricity was used.

The transportation considered the average distance between the production sites and the concrete plant and was about 100 km for glass and cement, 200 km for sodium hydroxide and 500 km for sodium silicate and carbonate. These distances are based on averages of the minimum and maximum distances between the production centers of the different materials and the considered concrete plan.

## 3. Results

### 3.1. Compressive Strength

**Figure 2:**
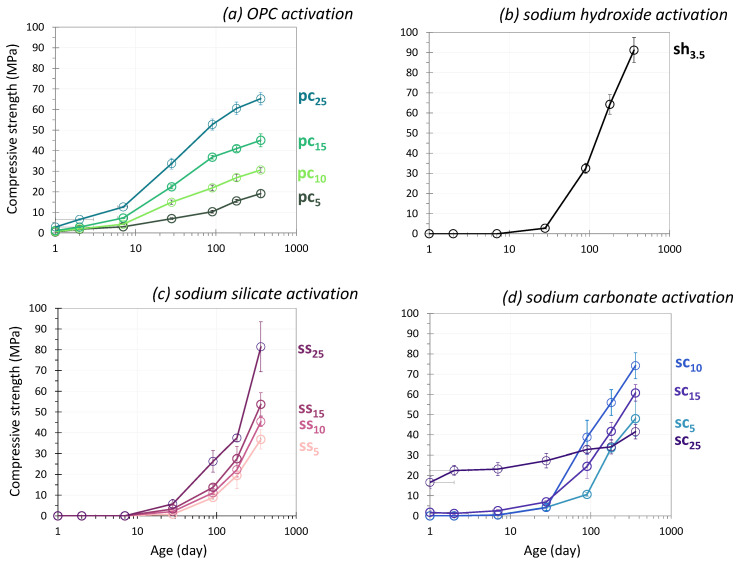
Influence of the nature and amount of the activator on the compressive strengths of activated glass cullet powder (20 °C) (logarithmic scale).

Figure 2 presents the compressive strength results of glass powder pastes activated with OPC (a), sodium hydroxide (b), sodium silicate (c) and sodium carbonate (d) at 20 °C.

#### 3.1.1. OPC Activation

Figure 2a shows the compressive strength results for glass powder pastes activated with different amounts of OPC. The general trend was: the greater the proportion of OPC in the paste, the higher the strength of the paste. At 1 day, the use of 15 wt% of OPC allowed the paste to reach 1 MPa. Lower amounts were associated with lower performance and samples were not easy to demold. Using 25 wt% of OPC allowed us to reach 3 MPa, which was quite similar to a ground granulated blast slag cement (20 wt% cement and 80 wt% slag) [51]. The use of 5 wt% of OPC allowed the strength to be measured at 2 days. At 7 days, the compressive strength of the four systems (pc_5_, pc_10_, pc_20_ and pc_25_) showed at least a twofold increase in strength (compared to the value at 2 days). At 28 days of curing, the pastes reached 7 and 34 MPa with OPC amounts between 5 and 25 wt%. These systems could therefore find applications in a variety of fields, depending on the performance requirements: road applications for the lowest performance, other applications for higher strength. From 90 to 360 days, compressive strength increased significantly, between 19 and 65 MPa, for pastes using between 5 and 25 wt% of OPC. A compressive strength of 60 MPa (180 days) would be widely conceivable for structural applications, provided that high strength at an early age was not required. The strength evolution was typical of a pure Portland cement, with a rapid increase in strength in the first few days of reaction, followed by a decrease in the kinetics of strength development afterward. This behavior was evident when the percentage of OPC was large. In contrast, for the systems containing 5 wt% of OPC, the strength development was gradual between 1 and 360 days, with strengths between 0.5 and 19 MPa. In the case of cement-activated glasses, strengths increased by 50% on average between 28 and 90 days and doubled between 28 and 360 days (Figure 2a), whereas Portland cements had generally reached about 80% of their strength at 28 days. These results suggest that the first strength gains were mainly due to the cement but that the glass then took over and improved the performance of the systems.

#### 3.1.2. NaOH Activation

Figure 2b presents the compressive strength results of glass powder paste sh_3.5_ activated with sodium hydroxide (3 mol/L) at 20 °C. For this system, no significant strength was observed up to 7 days of reaction (lower than 0.4 MPa) and then a gradual increase was seen up to 28 days, reaching 2.8 MPa at that age. Beyond 28 days, strength increased significantly, as the values were multiplied by 10 between 28 and 90 days and by 3 between 90 and 360 days. The system activated with sodium hydroxide differed from that activated with cement, with a significant improvement in strength even beyond 90 days: the strength increased by 0.38 MPa/day between 90 and 180 days, values that were much higher than those obtained with cement activation, which were less than 0.10 MPa/day, assuming a liner increase. At 360 days of curing, the strength increase was more moderate but still significant, allowing the system to reach strengths of around 90 MPa.

#### 3.1.3. Sodium Silicate Activation

Figure 2c shows the evolution over time of the compressive strength of pastes activated with different sodium silicate contents. The strength development was very different from that in systems activated with cement, which was characterized by a significant increase in strength in the first days and then a slowdown at later ages. The activation of the glass powder with Na-silicate was similar to hydroxide activation, with an initial phase during which the mechanical strength was very low, or even zero. During the first 7 days of curing at 20 °C, the strength was barely measurable, whatever the amount of Na-silicate used. The low performance was probably due to a slow attack of the siliceous network of the glass, related to the high modulus (relatively low alkali content) of the activator. The first significant compressive strength values were measured at 28 days of cure, and ranged from 1 to 6 MPa, increasing proportionally with the amount of Na-silicate. Beyond 28 days, the systems entered a phase where the strength increased in a very notable way. At 90 days, values were at least 5 times greater than those obtained at 28 days (for ss_10_, the strength increased from 2 MPa to 11 MPa). The activity of the glass appeared to be continuous; the increase in performance continued almost linearly with time and no plateau was reached, even after 360 days. The system with the highest Na-silicate content (25 wt%) had a larger deviation from the others. This high sodium silicate content provided the alkali needed to attack the silica network of the glass [52].

#### 3.1.4. Sodium Carbonate Activation

Figure 2d illustrates the compressive strength of glass powder pastes activated with different amounts of sodium carbonate. The evolution of strength development is divided into three stages:

- *Early age (0–7 days)*

For low activator contents (5 and 10 wt%), the strength was less than 1 MPa at 7 days. Demolding was not possible at 1 day and strength was not measurable at 2 days. The system containing 15% carbonate reached 1 MPa at 1 day of cure, and the strength continued to increase to 2.6 MPa at 7 days. The mixtures with 25% carbonate differed from the other systems, with a significant strength at 1 day of curing (17 MPa), which reached 22 MPa at 2 days and started to stabilize at 7 days.

- *Medium term (7–28 days)*

Similar moderate kinetics of strength development were seen with 5, 10 and 15% of activator, with strength not higher than 10 MPa at 28 days. At 25% carbonate, a strong slowdown in the development of strength was observed, with a value that stabilized at around 27 MPa. However, the strength remained the highest of the systems studied.

- *Long term (28–360 days)*

Systems with 5 and 15% carbonate showed similar development with a significant increase at 90 days, continuing through 360 days of cure. Strength with 25% carbonate increased slowly to 41.5 MPa at 1 year of curing. For 10% carbonate, particular behavior was noted, where a significant increase in strength was observed after 28 days. From 90 days onwards, the system sc_10_ presented the highest strength and surpassed all the other systems.

### 3.2. Hydration Kinetics

Isothermal calorimetry was conducted to evaluate the potential of the activator used to activate glass powder. The reaction kinetics can also reflect the reactivity of the system, as shown by Zhang et al. [53].

#### 3.2.1. OPC Activation

**Figure 3:**
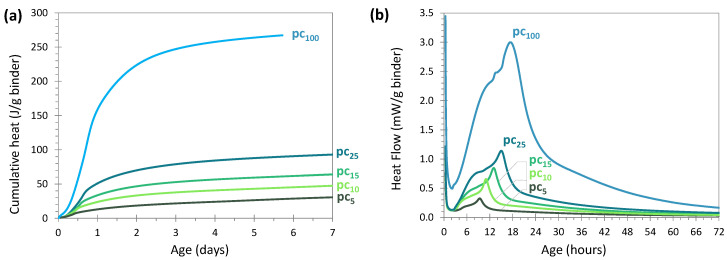
Cumulative heat (**a**) and heat flow (**b**) evolution of glass cullet powder activated with different amounts of cement CEM I 52.5N (20 °C, binder = glass + cement). Comparison with a paste containing only cement CEM I 52.5N.

Figure 3 presents the isothermal calorimetry analysis of the four systems based on glass powder activated with 5, 10, 15 or 25 wt% of CEM I 52.5N. A system with cement alone was used for comparison (red curve pc_100_). For all the pastes, the water/solid ratio was 0.30 and molding took place under the same conditions. Figure 3a shows the cumulative heat in J/g of binder (binder = glass + cement) during the first 7 days of reaction, and Figure 3b presents the heat flow in mW/g of binder during the first 72 h of reaction.

##### Behavior of the Systems With and Without Glass

The curves of the systems with glass in Figure 3a,b show similar patterns to those of cement alone (pc_100_), but with characteristic intensities and times that differ according to the cement content. Thus, on the one hand, rapid heat release kinetics were observed during the first 24 h, followed by strong slowdowns thereafter (Figure 3a). On the other hand, the thermal events characterized by heat flow peaks were almost the same but were delayed in time (Figure 3b). The similarity of the curves with and without glass suggests that it was, essentially, the hydration of cement that was measured, but that its intensity and kinetics were modified by the presence of glass. It should be noted that a thermal contribution by the reaction of glass is not to be excluded and may have been made possible from an early age thanks to the alkalinity brought by the cement. Nevertheless, some works have shown that the pozzolanic reaction of glass occurs later and releases less heat than cement [54].

#### 3.2.2. Sodium Hydroxide Activation

**Figure 4:**
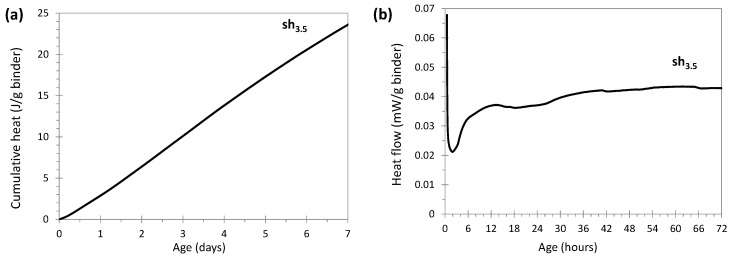
Evolution of cumulative heat (**a**) and heat flow (**b**) in glass cullet powder activated with sodium hydroxide 3 mol/L (20 °C, binder = glass + NaOH).

Figure 4a,b show the cumulative heat in J/g of binder (binder = glass + NaOH) and the heat flow in mW/g of binder, respectively, in the system sh_3.5_ activated with sodium hydroxide. This system presented a slow but constant heat increase until 7 days of reaction (Figure 4a), probably associated with the reactivity of the glass in the paste during the first few hours. However, unlike an OPC-glass system, the system sh_3.5_ showed no sharp increase during the first hours and the heat measured was modest (<25 J/g of binder). No distinctive heat peak was seen for NaOH activation compared to OPC systems. The calorimetry results probably explain why no significant compressive strength was detected in the early days of the reaction process, assuming that the reaction providing strength development was exothermal. However, it is worth noting that the complexity of the reaction mechanisms, such as Si-Al-Si polymerization and hydration, in low-calcium alkali-activated binders suggests that other factors may also influence strength development. This diversity of reactions can lead to varied strength development profiles, highlighting the need for a thorough understanding of the interactions among the different components of the mixture.

#### 3.2.3. Sodium Silicate Activation

**Figure 5:**
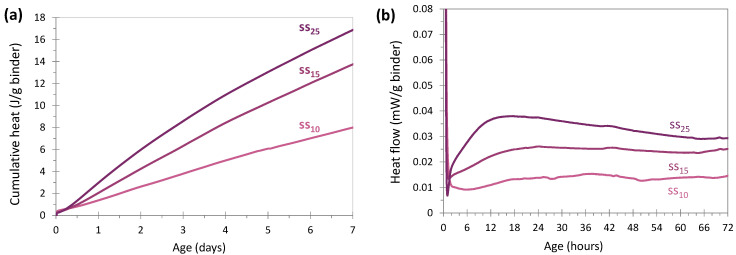
Evolution of cumulative heat (**a**) and heat flow (**b**) of glass cullet powder activated with different amounts of sodium silicate (20 °C) (binder = glass+ (dry) sodium silicate).

Figure 5a shows the cumulative heat in J/g of binder (binder = glass + (dry) sodium silicate) during the first 7 days of reaction for the systems activated with sodium silicate, and Figure 5b presents the heat flow in mW/g of the same binder during the first 72 h of reaction. The paste with 5 wt% of sodium silicate (ss_5_) is not shown because the heat release was very low and not detected by the device.

##### Behavior of the Glass and Sodium Silicate Systems in Comparison with Cement Activation

The cumulative heat curves with 10, 15 and 25 wt% of silicate increased progressively with time and with the quantity of activator. This evolution was very different from what was observed for cement activation, which was characterized by a rapid, significant increase in heat in the first hours, then by a stabilization until the end of the measurement (7 days). When sodium silicate was used, the progressive increase in heat was similar to that obtained with sodium hydroxide.

The heat release during the first 7 days increased with the silicate content in the systems. This indicated that more sodium silicate promoted a faster and greater reaction. However, the values obtained, even with the highest silicate content (25 wt%), remained quite low (below 18 J/g of binder at 7 days, compared to 25 J/g of binder for activation with 3.5% NaOH or 250 J/g for CEM I 52.5N cement), which explains the fact that mechanical strengths were not detected by the device at an early age.

##### Effect of the Amount of Sodium Silicate on the Heat Flow

Generally, in the case of sodium silicate activation, the curves obtained by calorimetry are characterized by two phases, as explained by Sagoe-Crentsil [55], who worked on systems composed of metakaolin activated with sodium silicate. The first phase presents a significant heat release during a rather short period of time. In this study, this phase is associated with the beginning of the dissolution reaction and the hydrolysis process. During the longer, second phase, the heat flow is more moderate. In the case of slags, this phase is explained by the formation of the first reaction products [56]. In our case, the main peak of phase 2 was not well-defined and was of low intensity. This could be explained by the structure of the glass, which was more polymerized and harder to attack than slags [57]. The increase in the peak intensity with the amount of silicate indicated that the addition of activator allowed the more efficient dissolution of the glass.

#### 3.2.4. Sodium Carbonate Activation

**Figure 6:**
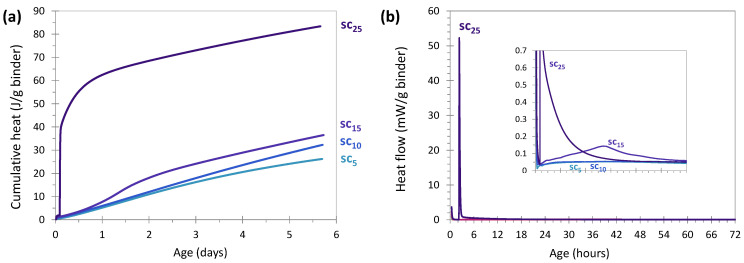
Cumulative heat (**a**) and heat flow (**b**) evolution of glass cullet powder activated with different amounts of sodium carbonate (20 °C) (binder = glass + Na_2_CO_3_).

Figure 6a,b show the cumulative heat in J/g binder (binder = solid = glass + Na_2_CO_3_) and heat flow in mW/g binder, respectively, for sodium carbonate activation during the first 6 days of reaction. In Figure 6a, the cumulative heat increased with the amount of activator. The glass paste activated with 25 wt% showed very high cumulative heat from the early hours of reaction, reaching a value of 40 J/g after 3 h. The heat releases of the other systems were quite similar, with values ranging from 23 to 33 J/g after 5 days of reaction. The high heat release with 25% carbonate (also visible in Figure 6b, with a very large peak of 50 mW/g) was probably related to the high carbonate content used in the system. A super saturation in carbonate led to a strongly exothermic hydration of the activator, via the passage from an anhydrous form of sodium carbonate to a hydrated form [58].

The absence of these peaks for the systems with 5 and 10% activator could be explained by the greater difficulty in activating glass compared to slag. In addition, activation with carbonate is known to be long compared to other activators (silicate or hydroxide) [59]. Decreasing the amount of activator decreased the intensity of the peak and also delayed its appearance.

## 4. Environmental Impact

Alkali-activated materials are often presented as environmentally friendly materials so, in order to study the environmental interest of the different systems, a life cycle assessment was carried out. In this study, the functional unit was defined as “producing 1 m^3^ of activated glass cullet pastes”.

### 4.1. LCA for 1 m^3^ of Activated Glass Cullet Pastes

The objective was to establish the environmental impact of different alkali-activated glass-based systems, to compare these materials with one another and also to make comparisons between the different constituents used.

#### 4.1.1. Contribution of the Different Constituents

**Figure 7:**
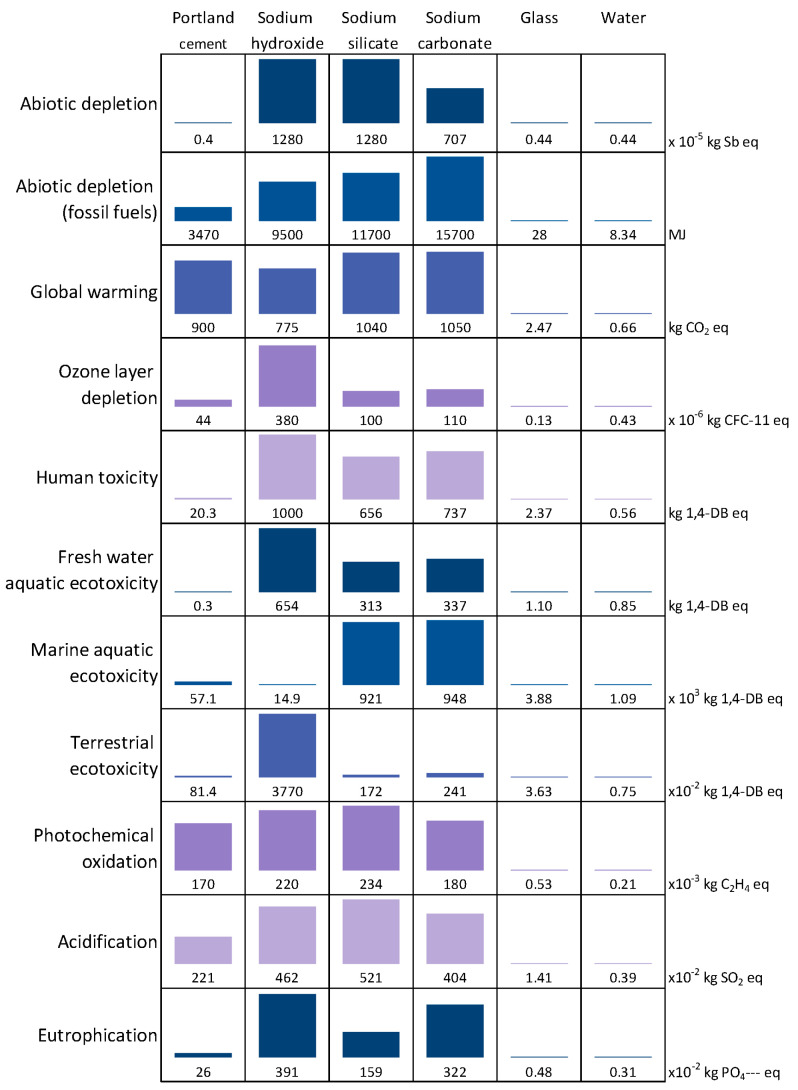
Impacts of the manufacture of 1 ton of constituent (without transport).

Figure 7 presents the impact of 1 ton of the constituent parts (cement, sodium hydroxide, sodium silicate, sodium carbonate, glass and water) on the 11 indicators chosen. The transportation was not included in order to consider only the manufacture of the constituent. Globally, glass and water have the lowest impact because glass is considered a re-used material and thus the impact of its manufacturing is zero. Sodium silicate, hydroxide and carbonate have the highest impacts, explained by their energy-intensive manufacturing process. The process of producing solid sodium silicate involves the reaction of sand and sodium carbonate at over 1400 °C [49]. The cement has an intermediate value. Figure 7 also shows that, for a given constituent, the contribution depends on the indicators studied. For instance, when sodium hydroxide is used, indicators related to toxicity and ecotoxicity (*human toxicity* and *fresh water*, *marine aquatic* and *terrestrial ecotoxicity*) make a high contribution. This is linked to the various chemical products, e.g., mercury, sodium, barium, calcium chloride and hydrochloric acid, among others, that entered into the production of sodium hydroxide [60] in the electrolysis phases.

Cement has less impact on *abiotic depletion*, due to the fact that cement and the raw resources from which it was made are considered to be present in almost infinite quantities on Earth. Conversely, the *abiotic depletion* indicator linked to the resource *fossil fuels* is more sensitive to the presence of cement, due to the need for significant heating of the raw materials to manufacture the clinker.

The silicate manufacturing process [49], with its melting, dissolving and filtration stages, consumes electricity, fuel oil and coal, and thus has significant environmental impacts. The energy inputs of the manufacturing process are expressed by the global warming indicator. Emissions to air include exhaust gas from the reaction furnace and NOx (oxides of nitrogen) emissions.

The NO_x_ emitted contribute not only to global warming, but to the formation of acid rain, eutrophication and the formation of photochemical ozone.

The carbonate manufacturing process [61], through its filtration, calcination and distillation stages, consuming electricity, fuel oil and coal, has significant environmental impacts. Air emissions include the gas released by the reaction furnace and are reflected by the *global warming* indicator.

#### 4.1.2. Comparison of the Impacts of the Different Systems

**Figure 8:**
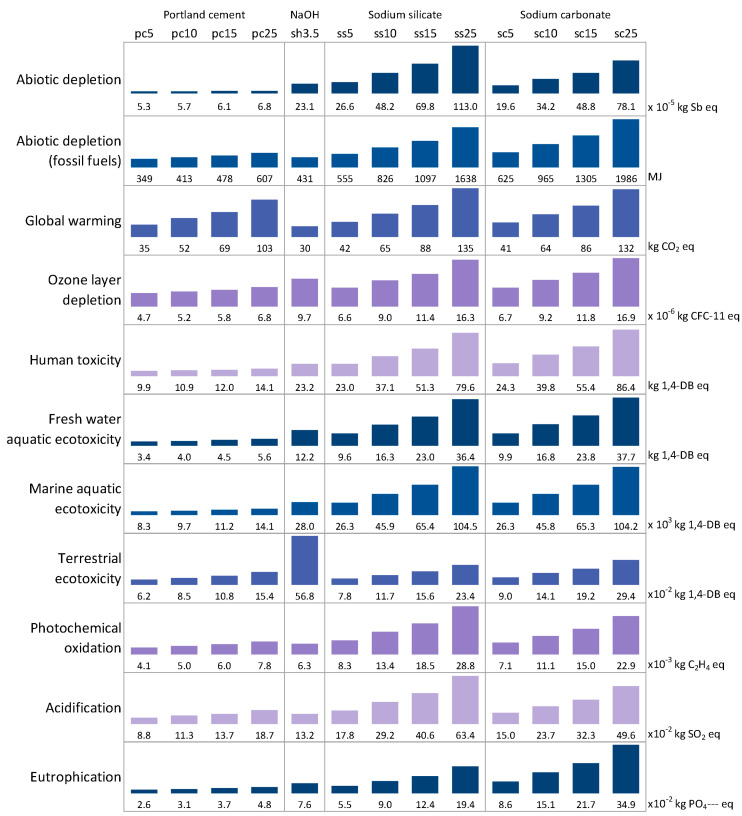
Impacts for 1 m^3^ of glass cullet pastes activated with different natures and proportions of activator (20 °C).

Figure 8 presents the values of the 11 indicators for glass cullet pastes activated with cement, sodium hydroxide, sodium silicate or sodium carbonate (5, 10, 15 or 25 wt%). For each indicator, the values of the systems have been classified as a histogram, in order to see the lowest and the highest impact for the indicator studied. For instance, for global warming, the systems containing 25 wt% of sodium silicate and 25 wt% of sodium carbonate were associated with higher impacts when compared to the other pastes.

The results show that the systems containing the highest percentage of activator are associated with the highest impact, especially for sodium silicate and carbonate. When the performances of the systems are not considered, using cement as activator seems to be more environmentally friendly than using other pastes (except for the impact on global warming).

Furthermore, it was important to also consider the mechanical performance because, in the final applications, we were looking for a level of performance, and not a volume of the system. This is reflected here through the study of the Performance Impact Indicator relating to global warming.

### 4.2. The Performance Impact Indicator

The previous section compared the environmental impacts linked to the production of 1 m^3^ of activated glass paste with activators of different natures. To account for the functioning of these systems, an indicator called the Performance Impact Indicator (PII) was chosen for use. As explained by Damineli et al. [62], this makes it possible to compare concretes with different performance levels. It is defined as the value of a given environmental indicator, linked to the production of 1 m^3^ of paste, divided by the mechanical strength of this same system. The environmental indicator used here was global warming.

Global warming has the advantage of being known to the general public, and its calculation is recognized as being robust and reliable. Its evaluation is considered to be consistent regardless of the method used. The unit of the PII is then the quantity of equivalent CO_2_ emitted by the production of 1 m^3^ of paste per MPa of mechanical strength recorded by this system (kg of CO_2eq_/m^3^/MPa) (Equation (1)).

(1) $$PII\;paste=\frac{Global\;warming/m^{3}\;paste}{Compressive\;strength\;paste}[\frac{\frac{{kgCO}_{2eq}}{m^{3}}}{MPa}]$$

**Figure 9:**
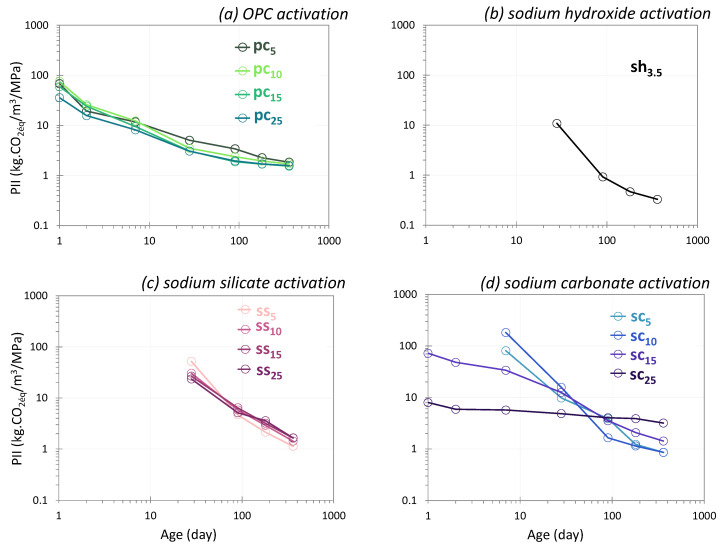
Performance Impact Indicator (PII) of glass cullet powder activated with different activators between 1 and 360 days (logarithmic scale).

**Figure 10:**
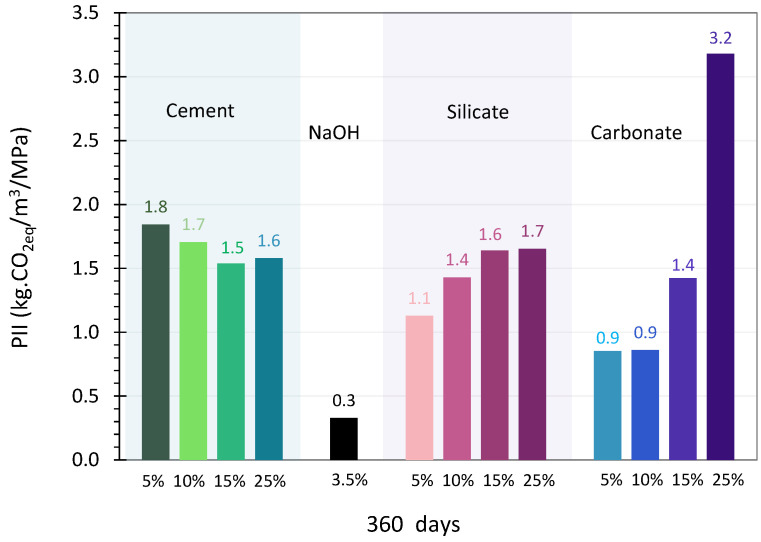
Performance Impact Indicator (PII) of glass cullet powder activated with different activators at 360 days.

The PII of the systems activated with different types of activators between 1 and 360 days are presented in Figure 9, and the final values (at 360 days) are in Figure 10.

#### 4.2.1. OPC Activation

Figure 9a plots the evolution of this indicator against time for glass activated with cement (5, 10, 15 and 25 wt%). The values decrease over time, which can be explained by the increase in compressive strength between 1 and 360 days (while the CO_2_ impact remains constant). At one day, the glass activated with 25 wt% of cement had the lowest PII (34 kg CO_2eq_/MPa, while the other activators gave values between 56 and 72 kg CO_2eq_/MPa). For this system, the strength obtained at this time (3 MPa) made it possible to compensate for the large quantity of CO_2_ emitted. Conversely, with less cement (5, 10 and 15 wt%), the strengths obtained at one day (less than 1.2 MPa) did not make it possible to counterbalance the CO_2_ of the cement.

From two days onward, the significant increase in performance for all the systems enables the PII to be significantly reduced. Using 25 wt% of cement still has the lowest impact (it has the highest compressive strength). At 28 days, the increase in strength reduces the value of the impacts, which all become less than 5 kg CO_2eq_/MPa. At 180 days and up to 360 days, the impacts tend towards similar values, varying between 1.8 and 1.5 for 5 and 25 wt% of cement, respectively.

The environmental impacts related to the presence of cement increase significantly with the amount of cement in the system (see Figure 9a), which is consistent as cement has a significant environmental impact.

#### 4.2.2. Sodium Hydroxide Activation

For sodium hydroxide activation, the Performance Impact Indicator (PII) relating to global warming was calculated from 28 days because, before this time, the pastes did not have any strength (Figure 9b). The initial value of the PII was 11 kg CO_2eq_/MPa and it decreased over time. The significant improvement in performance permitted a value of less than 1 kg CO_2eq_/MPa to be obtained from 90 days onward, dropping to 0.3 kg CO_2eq_/MPa at 360 days.

After 90 days of curing, sodium hydroxide activation always gave a lower PII than activation with cement, regardless of the proportion used.

#### 4.2.3. Sodium Silicate Activation

For sodium hydroxide activation, the Performance Impact Indicator was calculated from 28 days because, before this time, the pastes did not have any detectable strength (Figure 9c).

At 28 days, using 5 wt% of sodium silicate (ss_5_) gave the highest value (52 kg CO_2eq_/MPa), since it was this system that presented the lowest performance. At this time, the higher the level of activator, the lower the PII. This can be explained by the fact that the systems that contain the most silicate also have the best strengths, which makes it possible to compensate for the significant amount of CO_2_ in the system.

From 90 days onward, the significant increase in strength significantly reduces the PIIs, which all become less than 7 kg CO_2eq_/MPa. In the longer term, the PII continues to decrease to reach values below 2 kg CO_2eq_/MPa.

At 360 days, these values are between 1.1 and 1.7 kg CO_2eq_/MPa for the two extreme systems (5 and 25 wt% of sodium silicate). These values are similar to those obtained for cement-activated glass, which are between 1.8 and 1.5 kg CO_2eq_/MPa.

#### 4.2.4. Sodium Carbonate Activation

Figure 9d presents the evolution of the Performance Impact Indicator (PII) relating to global warming from one day for systems with 15 and 25 wt% of sodium carbonate and from seven days for those with 5 and 10 wt%. For the latter, no strength was detected before seven days. From one day, the PII of the system with 25 wt% of sodium carbonate was the lowest (8 kgCO_2eq_/MPa). Despite a large quantity of activator, the 16 MPa of mechanical performance recorded by this system did not counterbalance the CO_2_ emitted, unlike the system with 15 wt% of carbonate, for which the strength value was low (1.2 MPa). At two days, although the trend remained the same, the increase in performance decreased the PII of the two systems (15 and 25 wt%). From seven days, the PII was calculated for the four systems. Its value remained low for sc_25_, with the highest activator content (25 wt%), and stabilized at 4 kg CO_2eq_/MPa as the strength stabilized over time. For the activator contents of 5 and 10 wt%, the low strengths (less than 1 MPa) obtained at an early age do not counterbalance the CO_2_ attributed to the carbonate.

The PII values were then very high, especially for the system with 10 wt%, which was 177 kg CO_2eq_/MPa. At 28 days, a very significant drop in the PII was observed, explained by a notable gain in performance from this point. The strength obtained compensated for the value of the indicator, even though the strength of the systems at 5 and 10 wt% was low (4 MPa). These results showed a strong environmental potential from the moment the first mechanical performances appeared. From 90 days onward, using 10 wt% of activator led to the lowest PII value. Over time, and thus with increasing strength, the value continued to decrease to reach 0.7 kg CO_2eq_/MPa for the lowest levels of activator (5 and 10%). For glass activated with 15 and 25%, the calculated impacts were, respectively, 1.5 and 3.3 kg CO_2eq_/MPa.

After one year of curing at 20 °C, the PIIs of systems with 5 and 10 wt% were less than 1 kg CO_2eq_/MPa. They are lower than those obtained with cement or sodium silicate but higher than with hydroxide (0.3 kg CO_2eq_/MPa).

## 5. Discussion

The compressive strength of all glass systems activated with different activators at 1, 2, 7, 28, 90, 180 and 360 days is summarized in Table 5, where the color used corresponds to a value of compressive strength (red for low performance and green for high). By comparing the values of all the systems, it can be seen that the strength development differed depending on the nature of the activator used. For sodium hydroxide, sodium silicate and sodium carbonate, no significant strengths were observed before seven days of curing (20 °C) because the glass needed time to react. The results are in accordance with Zhang et al. [28], showing that it is difficult for waste glass to react with alkaline solutions due to its stable physicochemical properties. Thus, if early performance is needed, it would be preferable to use cement or sodium carbonate (25%) instead of sodium hydroxide or sodium silicate. Cyr et al. [23] mixed GCP with a fineness of 4170 cm^2^/g and cured at 20 °C with a 5 M sodium hydroxide solution, obtaining a compressive strength of 5 MPa at 28 days. This result was close to the value presented in this study, with sh3.5, which was around 3 MPa (Table 5). The higher compressive strength in Cyr et al.‘s study can be attributed to the higher concentration of sodium hydroxide and the finer glass powder used.

However, the activators associated with the highest compressive strength in the short term (as with OPC activation) did not show the highest compressive strength in the longer term, which means that, to obtain high performance in the long term, these types of activators are not preferable. With OPC activation, the performance was measurable at one day of curing (20 °C) but, at 360 days, cement activation was not associated with the highest compressive strength; sodium hydroxide activation proved to be better at that age. This observation is consistent with the findings of Torres-Carrasco and Puertas [63], who observed similar trends with silicate- and sodium hydroxide-activated systems. The same behavior was observed for the system with 25 wt% of sodium carbonate, which had a high value at one day of curing but no further evolution and a compressive strength of 42 MPa measured at 360 days of curing. The nature and amount of activator seems to mainly impact long-term performance. From 90 to 360 days, sodium hydroxide and sodium carbonate were the two activators associated with the highest compressive strength but with the lowest amount of the activator. At 180 days of curing, the highest compressive strengths were obtained for the systems using 10 wt% of sodium carbonate or 4 wt% of sodium hydroxide, with values of 56 and 65 MPa, respectively. Globally, the reaction kinetics of glass are slow because glass has a polymerized structure, making it harder to attack. For silicate activation, the higher the amount of activator, the higher the compressive strength. This behavior has been corroborated by Torres-Carrasco and Puertas [26], who demonstrated that compressive strength performance was better at higher alkaline concentrations with sodium silicate activation. Cheng et al. [34] also showed that higher activator content improves long-term performance. It should be noted that the lower performance of systems with low activation could also be due to excess water in the system. In this study, the water/binder ratio was constant, but the water/activator ratio differed for each activator level. However, to ensure a fairer comparison, adjusting the water dosage to maintain a constant paste flow rate could be considered. This would help mitigate the impact of varying water-to-activator ratios on the performance evaluation of different activation systems.

**Figure 11:**
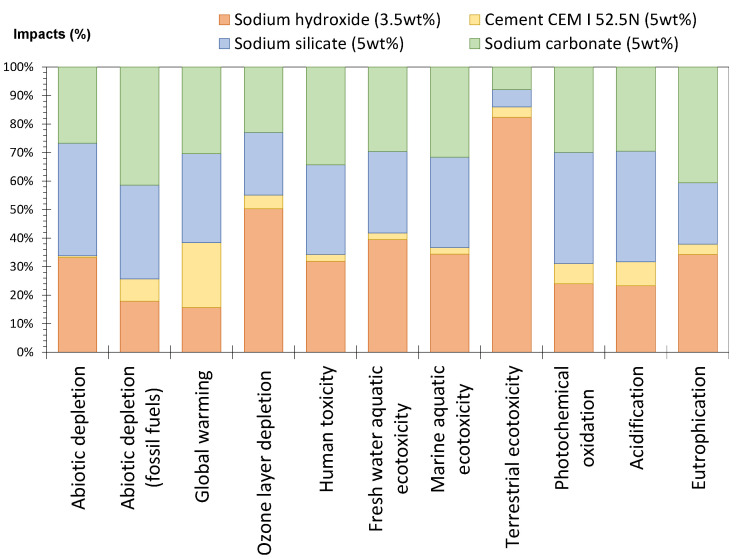
Impacts for 1 m^3^ of glass cullet paste with the activators used, with 5 wt% by mass and 3.5 wt% for sodium hydroxide.

The presence of the activator, necessary for alkali-activation, has a non-negligible effect on the environmental impact of the paste. As shown in Section 3.1, regardless of its nature, the environmental impact of the system was predominantly attributable to the activator. Figure 11 presents the impact of the four activators (used at 5 wt% except for sodium hydroxide, which was used at 3.5 wt%) on various indicators. The results show that sodium hydroxide (even used in the lowest amount compared to other activators) is globally associated with the highest impact. This is consistent with the results of Turner and Collins [64], who observed that sodium hydroxide production is energy-intensive which contributes significantly to its overall environmental footprint.

This means that, in a comparison of pastes, using sodium hydroxide was less environmentally friendly but, with time, this system showed the best compressive strength. For this reason, performance should be taken into account when assessing the impact of systems, as reflected in the Performance Impact Indicator (PII).

The life cycle assessment (LCA) presented in this study highlights methodological limitations, particularly regarding the choice of the functional unit. While the stated objective was the production of 1 m^3^ of alkali-activated material, this approach may seem unconventional compared to the functional unit typically used in the concrete industry, namely, the volume of poured concrete. Additionally, comparing materials based solely on their intrinsic strength may be perceived as biased, as it does not fully consider the real-world performance of the materials under actual usage conditions. Nevertheless, the introduction of the Performance Impact Indicator (PII), which correlates the environmental impact of a formulation with its mechanical performance, has helped mitigate this limitation. This approach aligns with Damineli et al. [62], who recommended linking performance with environmental impact for a more comprehensive evaluation.

**Figure 12:**
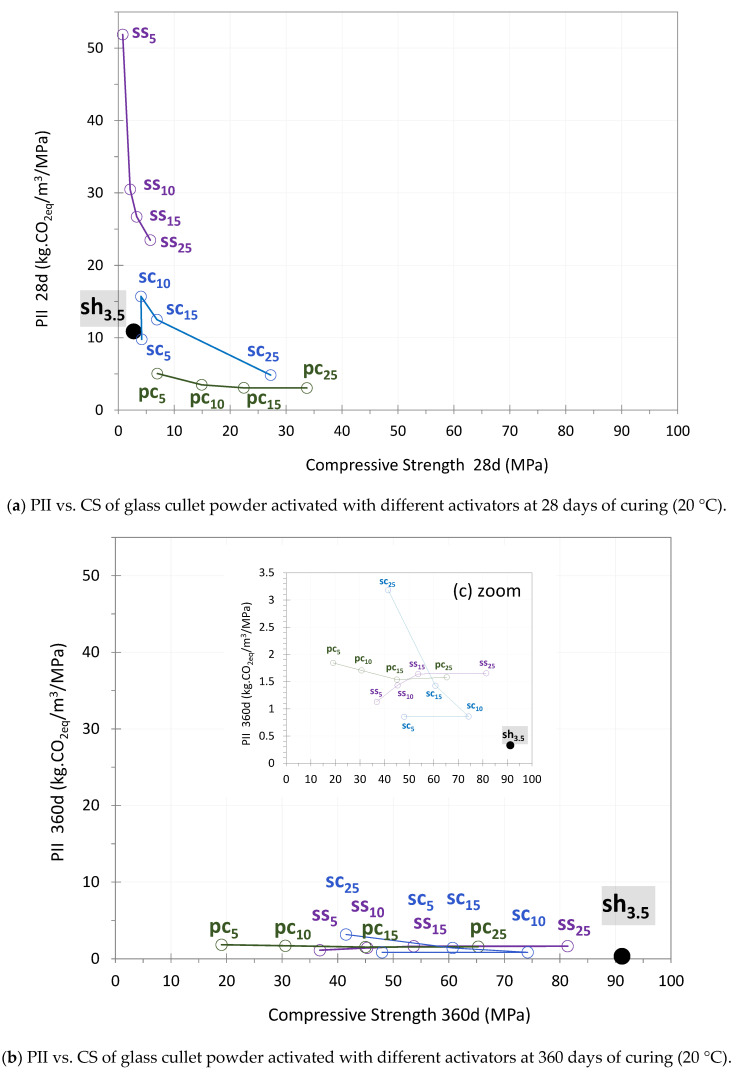
Performance impact indicator (PII) vs. Compressive Strength (CS) of glass cullet powder activated with different activators at 28 and 360 days of curing (20 °C).

Figure 12a,b present the Performance Impact Indicators (PIIs) of the systems against the compressive strength at 28 days and 360 days of curing (20 °C), respectively. Figure 12c provides a zoomed-in view of Figure 12b at 360 days of curing. In their study, Damineli et al. [62] presented the PII as a function of the compressive strength of various concretes after 28 days of curing. In this research, paste systems were studied instead of concrete. According to Damineli et al. [62], the best performing concretes are characterized by a position in the chart where high compressive strength coincides with a low PII. The results in this study show that, at 28 days of curing (Figure 12a), the data points are concentrated in the left area, associated with low compressive strength and a high PII. At early ages (0–28 days), the PII was lower when the activator content was higher. For example, in the case of sodium silicate (as seen in Figure 12a), the higher activator content resulted in an improvement in strength, which compensated for the CO_2_ emitted during its production and use. The study of compressive strength over time (Section 3.1) revealed that the performance of glass systems continues to improve beyond 28 days. As a result, the overall increase in strength (across all types and concentrations of activators) leads to a reduction in the PII. By 360 days, the PII was below 1 kg CO_2eq_/MPa when sodium hydroxide was used and less than 7 kg CO_2eq_/MPa for the other activators (Figure 9). Figure 12b presents the PII versus compressive strength at 360 days of curing (20 °C). Unlike at 28 days (Figure 12a), the data points are now located in the upper-right area of the chart, indicating higher compressive strength and lower PIIs. A closer look at the performance at 360 days is provided in Figure 12c, where the PII values range between 0 and 3.5 kg.CO_2eq_/m^3^/MPa. The systems activated with 3.5 wt% of sodium hydroxide (sh_3.5_) showed the best environmental and mechanical performance, positioned with both high compressive strength and low PIIs. With the exception of cement, the lowest PII values were achieved with the lowest levels of activator content. The system containing 10 wt% of sodium carbonate (sc_10_) also demonstrated good performance, with high compressive strength (the third-best value after ss_25_) and a low PII (the second-lowest value). At 360 days of curing (Figure 12c), using 25 wt% of sodium carbonate resulted in the highest PII (3.2 kg CO_2eq_/m^3^/MPa), while activation with cement or sodium silicate produced similar and lower PIIs of 1.8 and 1.7 kg CO_2eq_/m^3^/MPa, respectively. Using 5 wt% of sodium silicate resulted in a PII of about 1.1 kg CO_2eq_/m^3^/MPa. Overall, systems with high compressive strengths and relatively low activator content (below 25%), particularly sodium carbonate and sodium hydroxide, showed the lowest PII values, around 0.9 and 0.3 kg CO_2eq_/MPa, respectively. This demonstrates that systems with these activators offer a favorable balance between mechanical performance and environmental impact.

## 6. Conclusions

This study aimed to select the appropriate activator for the alkali-activation of soda–glass precursors, through the means of an analysis based on short- and long-term strength, reaction kinetics and life cycle assessment. Four different systems—Portland cement, high modulus sodium silicate, sodium hydroxide and sodium carbonate—were used at different proportions and at room temperature. The following conclusions can be drawn:

- Glass cullet powder could be used as a single precursor at room temperature and with different types of activator to reach a strength compatible with engineering properties (more than 80 MPa), although it presents slow reaction kinetics due to its high stability in alkaline media.
- The short-term strength of activated glass is usually low, except when there is a high content of an activator such as cement or sodium carbonate. These activators are thus preferable to obtain strength at an early age (0–7 days), but have a significantly higher environmental impact.
- Long-term strength could reach very high values, especially with activators that are slow in the first few days (sodium hydroxide, sodium silicate and sodium carbonate). These systems achieved high performance while limiting the use of activator, which is of environmental and economic interest.
- In the long-term, sodium hydroxide and sodium carbonate offer the best compromise between mechanical performance and environmental impact.
- Depending on the performances needed (strength at an early age or longer term, environmentally friendly), the choice of the activator could be adjusted, allowing for a wide range of compositions for this material.

## Figures and Tables

**Table 1:** Fineness of glass powder and cement CEMI 52.5N.

	Blaine	D_10_ (µm)	D_50_ (µm)	D_90_ (µm)
Glass powder	3000 cm^2^/g	2.6	15.6	59.8
CEM I 52.5N	3850 cm^2^/g	-	-	-

**Table 2:** Chemical composition of the glass and cement CEM I 52.5N.

% Mass	SiO_2_	Al_2_O_3_	Fe_2_O_3_	CaO	MgO	Na_2_O	K_2_O	TiO_2_	SO_3_	LOI *
Glass powder	71.5	2.3	0.9	10.4	1.5	12.3	0.5	0.1	-	0.5
CEM I 52.5N	19.3	5.3	2.6	63.2	2.0	0.1	0.9	-	3.5	2.9

* LOI: loss on ignition.

**Table 3:** Compositions of the glass pastes activated with different activators. Fineness of the glass: 3000 cm^2^/g; curing temperature: 20 °C.

**Portland cement activation**	**pc_5_**	**pc_10_**	**pc_15_**	**pc_25_**
Cement (%)	5	10	15	25
Cement (g)	20	40	60	100
Glass (g)	400			
Water (g)	126	132	138	150
Water/solid *	0.30			
Water/cement	6.3	3.3	2.3	1.5
**Sodium hydroxide activation**	**sh_3.5_**			
NaOH (%)	3.5 (equivalent to 3 mol/L)			
NaOH (g)	14			
Glass (g)	400			
Water (g)	120			
Water/solid **	0.30			
**Sodium silicate activation**	**ss_5_**	**ss_10_**	**ss_15_**	**ss_25_**
Sodium silicate solution (%)	5	10	15	25
Sodium silicate solution (g)	20	40	60	100
Glass (g)	400			
Total water (g)	122	124	126	131
Water/solid ***	0.30			
**Sodium carbonate activation**	**sc_5_**	**sc_10_**	**sc_15_**	**sc_25_**
Sodium carbonate (%)	5	10	15	25
Sodium carbonate (g)	20	40	60	100
Glass (g)	400			
Water (g)	126	132	138	150
Water/solid ****	0.30			

Solid: * glass + cement; ** glass + NaOH; *** glass + dry sodium silicate solution (35% of the total sodium silicate solution); **** glass + Na_2_CO_3_.

**Table 4:** Information relative to data used in this study.

Process	Modulus Used	Data Sources	Assumptions
Production of glass	/	Laboratory estimation	Average over the power grinders
Production of cement	Portland cement (CEM I), production mix, at plant, EN 197-1 (location: RER)	Ecoinvent© Zürich, Switzerland	-
Production of sodium silicate	/	[49]	-
Production of sodium carbonate	Sodium carbonate from ammonium chloride production, at plant/GLO U	Ecoinvent© [50]	-
Production of sodium hydroxide	Sodium hydroxide, 50% in H_2_O, production mix, at plant RER	Ecoinvent© [50]	Consideration of the French electricity energy mix
Production of slag	/	Industrial data	-
Transport	Transport, lorry 16–32 t, EURO5/RER	Ecoinvent© [50]	Estimation on distances
Production of water	Tap water, at user/CH S	Ecoinvent© [50]	-

**Table 5:** Summary of the compressive strength of glass cullet powder activated with different activators between 1 and 360 days of curing (20 °C).

Time	pc_5_	pc_10_	pc_15_	pc_25_	sh_3.5_	ss_5_	ss_10_	ss_15_	ss_25_	sc_5_	sc_10_	sc_15_	sc_25_
1 d					ND	ND	ND	ND	ND	ND	ND		
2 d					ND	ND	ND	ND	ND	ND	ND		
7 d					ND	ND	ND	ND	ND				
28 d													
90 d													
180 d													
360 d													
ND	Not detected			<3 MPa		3–15 MPa		15–50 MPa		50–80 MPa		>80 MPa	

## Data Availability

The original contributions presented in the study are included in the article, further inquiries can be directed to the corresponding author.
